# Core Trainees' study budget and study leave: a survey exploring trainees' needs and understanding of local processes in place within the North West

**DOI:** 10.1192/bjo.2021.439

**Published:** 2021-06-18

**Authors:** Chirag Shroff

**Affiliations:** CAMHS, Burlington House, Alder Hey NHS Foundation Trust

## Abstract

**Aims:**

Health Education England launched a new system for study leave and study budget on 1st April 2018, in response to trainees' concerns regarding the previous system. According to this, Health Education England would manage the study leave budget through its local offices, making the process of accessing study ‘more transparent, equitable and streamlined' for all trainees. At the RAP Oversight Committee meeting of the North West Deanery in 2019, trainees' uncertainties over the process was discussed by the local reps. It was aimed that there was a need to gather information on trainees' needs and understanding of local processes in place by the deanery to access study leave and study budget

**Method:**

A cross sectional survey was sent out to all the trainees by the Core Trainees year 1 RAP rep. A total of 6 relevant questions were designed and sent out to the trainees, allowing them 2 weeks’ time to respond. There were a total of 66 trainees who were sent the survey. The guidance mentioned in the 2016 Gold guide was used for reference to ensure the questions are relevant.

**Result:**

Of the total of 66 trainees who were sent the survey, there were 48 respondents. The results indicated that all 48 responders preferred study budget and leave process explained at induction. 47 of 48 respnders sought access to their study budget, 46 of 48 responders sought use of budget for external course and exam fees fudning, 27 of the 48 responders struggled to get study leave and 27 of 48 responders felt the current leave process was not satisfactory with 14 opining that there was scope for improvemement.

**Conclusion:**

The responses were collated by the trainee rep as a PowerPoint presentation containing graphical representation of trainees’ views regarding their study leaves and budget access. The survey results were made aware to the local board at the subsequent RAP Oversight Committee meeting to seek response and new guidance. There is a new system in place for study leave and study budgets, overseen by Health Education England. Overall, the survery attempted to understand and collate trainees' wants and needs, in effect improving trainee experiences.

